# Cognitive Functioning and Psychosocial Outcomes in Adults with Complex Congenital Heart Disease: A Cross-sectional Pilot Study

**DOI:** 10.1007/s00246-023-03376-7

**Published:** 2024-01-23

**Authors:** Charlotte E. Verrall, Derek L. Tran, Nadine A. Kasparian, Tracey Williams, Vincent Oxenham, Julian Ayer, David S. Celermajer, Rachael L. Cordina

**Affiliations:** 1 The University of Sydney School of Medicine, Sydney, NSW Australia; 2 Heart Centre for Children, The Children’s Hospital at Westmead, Sydney, NSW Australia; 3 Clinical Research Group, Heart Research Institute, Sydney, NSW Australia; 4 Department of Cardiology, Royal Prince Alfred Hospital, Sydney, NSW Australia; 5 School of Sport, Exercise and Rehabilitation, University of Technology Sydney, Sydney, NSW Australia; 6 Heart and Mind Wellbeing Center, Heart Institute and Division of Behavioral Medicine and Clinical Psychology, Cincinnati Children’s Hospital Medical Center, Cincinnati, OH USA; 7 Department of Pediatrics, University of Cincinnati College of Medicine, Cincinnati, OH USA; 8 Kids Rehab, The Children’s Hospital at Westmead, Sydney, NSW Australia; 9 School of Psychological Sciences, Macquarie University, Sydney, NSW Australia; 10 Department of Neurology, Royal North Shore Hospital, Sydney, NSW Australia

**Keywords:** Adult congenital heart disease, Cognition, Psychosocial functioning, Psychological distress, Quality of life, Resilience

## Abstract

Adults with complex congenital heart disease (CHD) are at risk for cognitive dysfunction. However, associations between cognitive dysfunction and psychosocial outcomes are poorly defined. Between June and November 2022, we prospectively recruited 39 adults with complex CHD who completed a computerized cognitive assessment (Cogstate) and validated psychosocial scales measuring psychological distress, health-related quality of life (HRQOL), and resilience. Participants had a mean age of 36.4 ± 11.2 years. Over half (62%) were women, most (79%) had complex biventricular CHD, and 21% had Fontan physiology. Prevalence of cognitive dysfunction was greatest in the domains of attention (29%), working memory (25%), and psychomotor speed (21%). Adjusting for age and sex, Pearson partial correlations between Cogstate *z*-scores and self-reported cognitive problems were small. Participants who lived in the most disadvantaged areas and those with a below-average annual household income had lower global cognitive *z*-scores (*p* = 0.02 and *p* = 0.03, respectively). Two-thirds (64%) reported elevated symptoms of depression, anxiety, and/or stress. Small correlations were observed between psychological distress and cognitive performance. Greater resilience was associated with lower psychological distress (*r* ≥ −0.5, *p* < 0.001) and higher HRQOL (*r* = 0.33, *p* = 0.02). Our findings demonstrate that adults with complex CHD have a high risk of cognitive dysfunction, though may not recognize or report their cognitive challenges. Lower socioeconomic status may be an indicator for those at risk of poorer cognitive functioning. Psychological distress is common though may not be a strong correlate of performance-based cognitive functioning. Formal cognitive evaluation in this patient population is essential. Optimizing resilience may be a protective strategy to minimize psychological distress and bolster HRQOL.

## Introduction

The adult population living with complex congenital heart disease (CHD) is rapidly growing [1–3]. As long-term survival and clinical outcomes continue to improve, there is increasing focus on optimizing health-related quality of life (HRQOL) [4].

Neurodevelopmental delay and disability are common in infants with complex CHD [5–8] and can manifest as long-term cognitive deficits later in life [9–16]. Recent studies have demonstrated that broad cognitive dysfunction is observed in adults with complex CHD and is associated with CHD complexity [17–20]. Adults with CHD may also experience elevated psychological distress and have poorer social and educational outcomes compared with healthy aging adults [4], including challenges at school, the workplace, and in relationships [21]. Socioeconomic status may be a key determinant of neurodevelopmental and psychological outcomes [22–25] and combined these factors can have a significant impact on overall HRQOL [26–28]. Unlike in children with CHD, the impact of cognitive dysfunction on broader health and psychosocial functioning in adults with complex CHD is poorly characterized. Understanding associations between cognitive functioning and psychosocial factors is essential for guiding targeted intervention strategies to optimize long-term cognitive functioning and overall well-being. The aim of the current study was to characterize associations between performance-based measures of cognitive functioning and self-report measures of psychosocial and socioeconomic factors in adults with complex CHD. We hypothesized that poorer cognitive performance would be associated with lower socioeconomic status and HRQOL, and higher levels of psychological distress. Our secondary hypotheses were that greater resilience would be associated with lower levels of psychological distress and greater HRQOL.

## Methods

### Participants

Ethics approval was obtained from the Sydney Local Health District Ethics Review Committee (RPAH Zone; 2021/ETH01181) and all participants provided written informed consent. Individuals with complex CHD aged 18 years or older were prospectively recruited as part of the Congenital Heart Disease Fitness Intervention Trial (CH-FIT) [29]. Classification of complex CHD included patients with moderate to severe CHD as defined by European Society of Cardiology disease classification guidelines [30]. Participation took place at the Royal Prince Alfred Hospital in Sydney, Australia between June and November 2022. Individuals with severe verbal comprehension difficulties impacting the capacity to understand task instructions were not included. Broader exclusion criteria for CH-FIT are described by Tran et al. [29]

### Cognitive Assessment

Participants completed a computerized cognitive assessment called Cogstate (Melbourne, Australia). The Cogstate battery consisted of a series of tests measuring psychomotor speed, processing speed, attention, visual learning and memory, verbal learning and memory, working memory, executive function, and social-emotional cognition. The full battery took approximately 45 min to complete. Assessments were administered in-person by a trained research assistant in a quiet assessment room with minimal distractions. Raw scores were transformed to *z*-scores using age-matched normative data. Where relevant, *z*-scores were reversed so lower scores reflected poorer performance; *z*-scores were used in all analyses. A global cognitive composite score was generated by summing the primary outcome *z*-score from each test and dividing by the total number of tests. In accordance with Cogstate guidelines, *z*-scores between >1 and 2 SD below normative means were indicative of mild cognitive dysfunction and aligned with performance below the 16th percentile of functioning in a normative sample. Scores ≥2 SD below normative means were classified as severe cognitive dysfunction representing performance at or below the 2nd percentile of functioning compared to population norms [31]. In other clinical cohorts, a *z*-score of >1 SD below normative mean on Cogstate demonstrates optimal sensitivity for detecting mild cognitive decline [31].

Participants completed the Conners’ Adult ADHD Rating Scales Self-Report Short Version (CAARS S:S) [32] to assess behaviors and symptoms indicative of attention-deficit hyperactivity disorder (ADHD). The CAARS S:S generates an ADHD Index and four factor-derived subscales; (1) Inattention/Memory Problems, (2) Hyperactivity/Restlessness, (3) Impulsivity/Emotional Lability, and (4) Problems with Self-Concept. Raw scores were converted to *t*-scores using age- and sex-normative data. A cut-off *t*-score of ≥65 (i.e., ≥1.5 SD from the mean) indicated challenges consistent with ADHD. The Autism Quotient-10 (AQ10) [33] was used to assess symptoms indicative of possible autism spectrum disorder. A cut-off score of >6 suggests a person should be considered for clinical assessment.

### Evaluation of Sociodemographic Factors

Sociodemographic factors were self-reported and included country of birth, primary language, marital status, residential address, living circumstance, highest achieved level of education, employment status, and gross annual household income. Geographical remoteness was determined using the Australian Statistical Geography Standard—Remoteness Classification [34]. Socioeconomic disadvantage was assessed using the Australian Index of Relative Socio-Economic Advantage and Disadvantage [35] which categorizes areas based on census information about the economic and social conditions of people and households within the area. Annual household income was categorized according to those with an annual household income below the Australian mean (i.e., ≤$120,000 Australian dollars) versus those with above (Australian Bureau of Statistics) [36].

### Evaluation of Psychosocial Factors

A lifetime psychological disorder referred to a person being diagnosed with a psychological condition at some time in their life [37].

*Depression, Anxiety and Stress Scales* (DASS-21, 21 items) assessed symptoms of psychological distress. Items range from 0 (“Never”) to 3 (“Almost always”). Higher scores indicate greater distress and were classified as normal, mild, moderate, severe, or extremely severe, based on the scale manual [38].

*Cardiac Anxiety Questionnaire* (CAQ) [39] includes 18 items that assessed cardiac-related anxiety. Items are scored on a 5-point Likert scale (0–4), with higher scores indicating greater anxiety.

*Impact of Events Scale—Revised* (IES-R) evaluated traumatic stress symptoms along three dimensions; intrusion, avoidance, and hyperarousal on a 5-point Likert scale (0–4), with all items anchored to congenital cardiac diagnosis. Higher scores indicate greater traumatic stress.

*Pediatric Quality of Life Inventory 4.0 Generic Core Scale* (PedsQL) was administered to evaluate HRQOL [40, 41]. The scale includes 23-items that encompass physical, emotional, social, and school/work functioning. Three summary scores are calculated (1) Physical Health Summary Score (2) Psychosocial Health Summary Score that comprises emotional functioning, social functioning, and school functioning and (3) Total Scale Score (HRQOL score) that encompasses all scores. Participants also completed the *PedsQL 3.0 Cardiac Module.* The cognitive problems score was included in the current analysis to measure self-reported cognitive functioning. Items on both the *PedsQL Generic Core 4.0 and Cardiac 3.0* scales are rated using a 5-point Likert scale, scores are reversed and transformed to scaled scores that range from 0 to 100, with higher scores representing greater HRQOL.

Resilience was measured using the *10-Item Conner-Davidson Resilience Scale* (CD-RISC-10). Items are scored on a 5-point Likert scale (0–4), with higher scores indicating greater resilience [42].

*The Coronavirus Anxiety Scale* [43] was included to account for anxiety that was experienced at the time of the study in response to the COVID-19 pandemic. Items are scored on a 5-point Likert scale (0–4), a total score ≥9 indicates probable dysfunctional coronavirus-related anxiety.

All questionnaires were completed within 1 month prior to completing the Cogstate assessment (mean 3.4 ± 7.3 days).

### Statistical Analysis

Data were analyzed using IBM SPSS Version 28 (IBM Corp). All data were evaluated for normality of distribution prior to analysis; normality was observed for all variables. Continuous variables were summarized using mean and SD and categorical variables were summarized using frequencies and percentages. Sociodemographic factors from the study cohort are qualitatively described in comparison to the latest Australian census population data (www.abs.gov.au/statistics).

Pearson partial correlation coefficient adjusting for age and sex as covariates was used to analyze the relationship between Cogstate *z*-scores and continuous variables. Correlation coefficients were interpreted according to published guidelines as small (*r* value between 0.1 and 0.29), moderate (0.3–0.49), and strong (≥0.5) [44, 45]. To analyze associations between Cogstate *z*-scores and categorical variables, two sample *t* tests and one-way ANOVA were used to compare means across levels of categorical data, mean difference and 95% confidence intervals (CI) are reported. Adjustment for multiple comparisons was not performed as this was an exploratory analysis. A *p* value of <0.05 was used to determine statistical significance.

## Results

### Participant Characteristics

Thirty-nine participants completed the Cogstate and psychosocial assessment. Sociodemographic and clinical characteristics are reported in Tables 1 and 2, respectively. No participants reported a previous diagnosis of stroke or brain injury. One participant had DiGeorge syndrome but scored within an average range on all measures of cognitive functioning. Thirty-five (90%) participants had completed ≥12 years of education which is identical to the rate reported in the general Australian population. Forty-two percent (*n* = 16) of the cohort had completed an undergraduate university degree or higher in comparison to 32% of the Australian population. Eleven (28%) participants reported that they had difficulties with learning at school; however, only 5 people (13%) had completed any formal evaluation to better understand these challenges. The rate of unemployment in our sample was higher than the national average (8% versus 3.5%, respectively) and four (10%) participants were receiving government-funded financial assistance relating to unemployment or disability.

**Table 1:** Sociodemographic characteristics

Variable	*n*	Result
Sex	39	
Female^a^ Male		24 (62%) 15 (38%)
Age, years; mean (SD)	39	36.4 (11.18)
Place of birth	39	
Australia Bangladesh Hong Kong Italy South Korea Syria		34 (87%) 1 (3%) 1 (3%) 1 (3%) 1 (3%) 1 (3%)
Ethnicity	39	
Arab Australian Bangladeshi Chinese German and Indian Italian Korean Vietnamese		1 (3%) 32 (82%) 1 (3%) 1 (3%) 1 (3%) 1 (3%) 1 (3%) 1 (3%)
Marital status	39	
Single, never married Defacto relationship Married Separated		14 (36%) 9 (23%) 15 (38%) 1 (3%)
Current living circumstance	39	
Living with relatives Living with partner Living with friends Living alone		16 (41%) 17 (44%) 3 (8%) 3 (8%)
Children	38^b^	
Yes No		15 (39%) 23 (61%)
Annual household income (Australian dollars)	25^b^	
≤ $120,000 > $120,000		8 (32%) 17 (68%)
Geographical remoteness classification	39	
RA1 (major cities) RA2 (inner regional)		36 (92%) 3 (8%)
Index of Relative Socioeconomic Advantage and Disadvantage		
1—Most disadvantaged area 2 3 4 5—Most advantaged area	39	5 (13%) 6 (15%) 7 (18%) 7 (18%) 14 (36%)
Highest grade of school	39	
Year 9/10 Year 11 Year 12		1 (3%) 3 (8%) 35 (90%)
Tertiary education	39	
Not Applicable TAFE, Graduate Diploma or Certificate Undergraduate Degree Master Degree Doctorate/PhD		9 (23%) 14 (36%) 10 (26%) 5 (13%) 1 (3%)
Employment	39	
Full-time employment (≥35 h per week) Part-time/casual employment (20–34 h per week) Studying and employed part-time Unemployed		25 (64%) 7 (18%) 4 (10%) 3 (8%)
Current disability benefits National Disability Insurance Scheme Disability Support Pension JobSeeker Payment Youth Allowance for JobSeekers Mobility Allowance Other	39	4 (10%) 3 (8%)^c^ 0 (0%) 1 (3%)^c^ 0 (0%) 0 (0%) 1 (3%)
Reported difficulties with learning at school? Yes No	39	11 (28%) 28 (72%)
Any assessment to understand your difficulties with learning?		
Yes No	38	5 (13%) 33 (87%)

*TAFE* Technical and Further Education

^a^One participant identified sex as female and gender as non-binary

^b^Reduced sample size reflects participants who did not provide this information

^c^One participant received funding through the National Disability Insurance Scheme and a JobSeeker payment

**Table 2:** Clinical characteristics

Variable	*n*	Result
Primary cardiac diagnosis	39	
Biventricular Repaired coarctation of the aorta or interrupted aortic arch Pulmonary stenosis (moderate or severe) Pulmonary atresia ASD, moderate to large and/or with associated abnormalities VSD with associated abnormalities and/or moderate or greater shunt Double outlet right ventricle Tetralogy of Fallot Transposition of the great arteries Fontan Circulation		31 (79%) 4 (10%) 5 (13%) 1 (3%) 4 (10%) 5 (13%) 1 (3%) 10 (26%) 1 (3%) 8 (21%)
Diagnosed attention disorder	39	2 (5%)
Diagnosed autism spectrum disorder	39	0 (0%)
Lifetime psychological diagnosis^a^ Depression Anxiety Post-traumatic stress disorder Other	39	16 (41%) 13 (33%) 9 (23%) 3 (8%) 0 (0%)
Previously or currently taken prescribed psychotropic medication associated with a psychological condition		
Yes No	16	7 (44%) 9 (57%)

^a^Psychological diagnoses are not mutually exclusive and several participants had more than one psychological diagnosis

### Cognitive Functioning

**Fig. 1:**
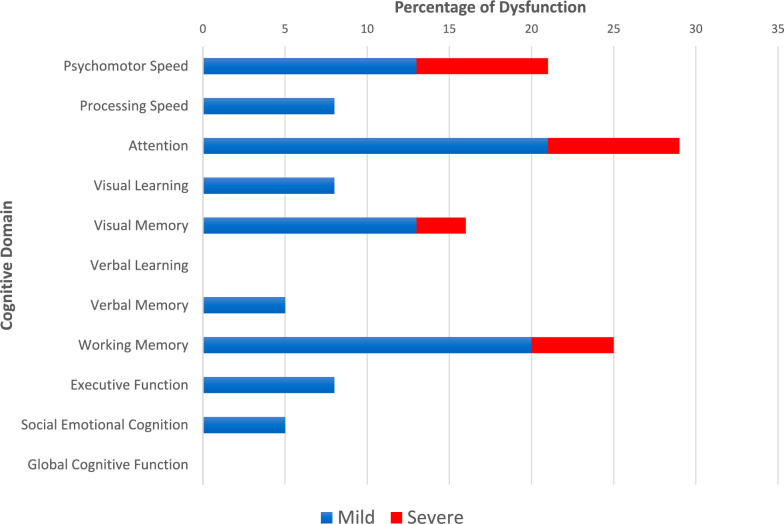
Proportion of mild and severe dysfunction in the cognitive domains assessed

Proportion and severity of cognitive dysfunction across the domains assessed are displayed in Fig. 1. Mean global cognitive composite *z*-score was −0.02 ± 0.46 and mean *z*-scores for psychomotor speed, attention, and working memory were below the normative mean, though within an average range (Table 3). While no participants had a global composite score within the ‘impaired’ range (i.e., *z*-score < −1), 8 (21%) participants exhibited dysfunction in ≥3 cognitive domains, indicating broad cognitive challenges. Women had a lower mean psychomotor speed *z*-score compared with men (−0.67 versus −0.12, respectively, *p* = 0.01), no other differences in cognitive functioning were observed based on sex. Participants with a Fontan circulation had lower mean processing speed *z*-scores compared with participants with complex biventricular CHD (−0.32 versus 0.16, respectively, *p* = 0.03). When grouped by complexity, participants with severe CHD had lower verbal memory *z*-scores compared with participants with moderate CHD (0.01 versus 0.55, respectively, *p* = 0.04).

**Table 3:** Proportion of scores exceeding cut-off thresholds on the Cogstate assessment, Conners Adult ADHD Rating Scale, and Autism Quotient-10

Cogstate		*n*	Whole group mean (SD)	Mild dysfunction		Mild group mean (SD)	Severe dysfunction	Severe group mean (SD)
Psychomotor speed^a^		39	−0.46 (0.77)	5 (13%)		−1.27 (0.38)	3 (8%)	−2.3 (0.19)
Processing speed^b^			0.06 (0.66)	2 (5%)		−1.2 (0.19)	0 (0%)	–
Attention^c^			−0.63 (0.9)	8 (21%)		−1.42 (0.17)	3 (8%)	−2.6 (0.33)
Visual learning^d^			0.36 (0.88)	3 (8%)		−1.16 (0.17)	0 (0%)	
Visual memory^e^			0.02 (0.95)	5 (13%)		−1.31 (0.34)	1 (3%)	−2.36 (–)
Verbal learning^f^			0.43 (0.58)	0 (0%)		–	0 (0%)	–
Verbal memory^g^			0.38 (0.75)	2 (5%)		−1.42 (0.08)	0 (0%)	–
Working memory^h^			−0.57 (0.83)	8 (20%)		−1.38 (0.24)	2 (5%)	−2.77 (0.96)
Executive function^i^			0.15 (0.86)	1 (3%)		−1.3 (–)	2 (5%)	−2.5 (0.37)
Social and emotional recognition^j^			0.03 (0.44)	2 (5%)		−1.3 (0.08)	0 (0%)	–
Global cognitive composite			−0.02 (0.46)	0 (0%)		–	0 (0%)	–

Conners Adult ADHD Rating Scale—Short Form					*Above Clinical Threshold* (*t*-score ≥ 65)			
ADHD Index		38			3 (8%)			
Inattention/Memory Problems					7 (18%)			
Hyperactivity/Restlessness					5 (13%)			
Impulsivity/Emotional Lability					2 (5%)			
Problems with Self-Concept					3 (8%)			

Autism Quotient—10 Item								
Symptom Score > 6		39			2 (5%)			

*ADHD* attention-deficit hyperactivity disorder

^a^Detection Test

^b^Groton Maze Chase Test

^c^Identification Test

^d^One Card Learning

^e^Groton Maze Learning—Delayed Recall

^f^International Shopping List Task (ISLT)

^g^ISLT—Delayed Recall

^h^One Card Back

^i^Groton Maze Learning

^j^Social Emotional Cognition Test

No participants had a diagnosis of autism spectrum disorder, and 2 (5%) participants had an existing diagnosis of an attention disorder. The proportion of participants that scored above the cut-off threshold on the CAARS S:S and AQ10 are reported in Table 3. Only one of the participants who scored above the clinical threshold on the ADHD Index had previously been diagnosed with an attention disorder.

### Psychological Outcomes

Sixteen (41%) participants reported a lifetime diagnosis of a psychological condition (50% of females and 27% of males), including depression (33%; 42% of females and 20% of males), anxiety (23%; 29% of females and 13% of males), and post-traumatic stress disorder (8%; 13% of females and no males). Of these, 8 (21%) had a comorbid psychological diagnosis (Table 2). Seventeen participants (94%) who had been diagnosed with a psychological condition had received support or therapy from a psychiatrist or psychologist and 7 (44%) had previously or were currently taking prescribed psychotropic medication associated with a psychological condition. Twenty-five (64%) participants reported currently elevated levels of psychological distress, including depression (26%), anxiety (48%), and stress (33%) (Table 4). Participants with a Fontan circulation reported significantly higher levels of anxiety compared to the biventricular complex CHD group (mean difference = −2.67, [95% CI = −5.02, −0.33], *p* = 0.01). When grouped by severity, no significant differences in levels of depression, anxiety, and stress were reported between those with moderate versus severe CHD. Mean resilience score indicated that the group scored in the lowest quartile compared to population norms (mean = 27.67 ± 6.47) [42]. No participants indicated elevated anxiety related to COVID-19.

**Table 4:** Psychosocial outcomes

Scale	*n*	Result
DASS-21		
Depression		
Normal	39	29 (74%)
Mild		4 (10%)
Moderate		5 (13%)
Severe		1 (3%)
Extremely severe		0 (0%)
Anxiety		
Normal	39	20 (51%)
Mild		7 (18%)
Moderate		7 (18%)
Severe		3 (8%)
Extremely severe		2 (5%)
Stress		
Normal	39	26 (67%)
Mild		6 (15%)
Moderate		5 (13%)
Severe		2 (5%)
Extremely severe		0 (0%)
CAQ Cardiac anxiety; mean (SD)	39	0.36 (0.96)
PedsQL Core; mean (SD)		
Total PedsQL score	39	73.02 (13.4)
Physical Health Summary Score		71.24 (16.85)
Psychosocial Health Summary Score		73.96 (14.04)
PedsQL Cardiac; mean (SD)		
Cognitive problems	39	62.56 (21.91)
IES-R Traumatic Stress Symptoms; mean (SD)		
Total score	39	0.48 (0.53)
Intrusion		0.51 (0.62)
Avoidance		0.5 (0.59)
Hyperarousal		0.42 (0.6)
CD-RISC 10—resilience; mean (SD)		
Total score	39	27.67 (6.47)
COVID-19 Anxiety Scale		
Total score ≥ 9	39	0 (0%)

### Associations Between Cognitive Functioning, Sociodemographic, and Psychosocial Factors

#### Cognition and Sociodemographic Factors

A significant difference in mean global cognitive composite *z*-scores was identified based on the Index of Socioeconomic Advantage and Disadvantage (*F* = 3.23(4, 34), *p* = 0.02); Tukey post hoc tests showed people living in the most disadvantaged areas had a lower global cognitive composite *z*-score compared with people living in the most advantaged areas (mean difference = −0.69, *p* = 0.02). Participants with an annual household income below the Australian average had a lower mean global cognitive composite *z*-score (mean difference = −0.42, [95% CI = −0.8 to −0.4], *p* = 0.03) and mean executive functioning *z*-score (mean difference = −0.64, [95% CI = −1.28 to −0.01], *p* < 0.05) compared with those with an annual household income above the Australian average. Lower visual learning *z*-scores were observed in participants who were single compared to those in a relationship (mean difference = −0.76 [95% CI = −1.3 to −0.22], *p* = 0.01) and participants who were living with parents or relatives compared to those living independently or with a partner (mean difference = −0.88, [95% CI = −1.58 to −0.18], *p* = 0.01).

Participants who had completed a university degree had higher mean *z*-scores in the global cognitive composite (mean difference = 0.29, [95% CI = −0.58 to 0.01], *p* = 0.03), attention (mean difference = 0.58, [95% CI = 1.15 to 0.004], *p* = 0.02), and visual learning (mean difference = 0.66, [95% CI = 1.21 to 0.09], p = 0.01) compared with those who did not have a university degree. Differences in Cogstate *z*-scores based on employment status were not evaluated due to small sample sizes.

#### Cognition, Psychological Factors, and HRQOL

Correlations between Cogstate *z*-scores and depression, anxiety, stress, and cardiac-related anxiety scores were small (Table 5).

**Table 5:** Pearson partial correlation coefficients between Cogstate *z*-scores and PedsQL, DASS-21, and CAQ scores (adjusted for age and sex)

	Cogstate											
	Global composite	Psychomotor speed	Processing speed		Attention	Visual learning	Visual memory	Verbal learning	Verbal memory	Working memory	Executive function	Social and emotional cognition
PedsQL Core												
Physical Health Summary Score	0.34*	0.23	0.22	0.27		0.37*	0.28	−0.03	0.05	0.22	0.11	0.24
Psychosocial Health Summary Score	0.18	0.16	−0.18	0.23		0.12	0.25	0.01	0.07	0.1	0.13	−0.03
Total HRQOL	0.27	0.21	−0.02	0.28		0.24	0.29	−0.01	0.07	0.16	0.13	0.08
PedsQL Cardiac												
Cognitive Problems	0.04	0.08	−0.23	0.13		0.14	0.01	0.12	−0.19	−0.01	0.12	−0.04
DASS-21												
Depression	0.29	0.22	0.25	0.19		0.06	0.23	0.06	0.18	0.23	0.14	0.22
Anxiety	−0.14	−0.004	0.21	−0.05		−0.21	−0.22	−0.04	−0.18	−0.10	−0.1	0.06
Stress	0.12	0.26	0.26	0.16		0.12	−0.11	−0.11	0.13	−0.02	−0.001	0.1
CAQ												
Cardiac Anxiety	−0.03	0.09	0.07	0.13		−0.27	−0.02	−0.15	−0.05	0.11	−0.12	0.04

*PedsQL* Pediatric Quality of Life Inventory, *DASS-21 *Depression, Anxiety and Stress Scale—21 Item, *CAQ* Cardiac Anxiety Questionnaire

* *p* < 0.05

Moderate correlations were observed between patient-reported physical health and global cognitive composite *z*-score (*r* = 0.34, *p* = 0.04) and visual learning (*r* = 0.37, *p* = 0.03). Correlations between patient-reported cognitive problems and Cogstate *z*-scores were small (Table 5). Moderate-to-strong negative correlations were identified between psychological distress and psychosocial health summary scores and HRQOL (*r* ≤ 0.35, *p* ≤ 0.01) (Table 6).

**Table 6:** Pearson partial correlations between PedsQL, DASS-21, and CD-RISC-10 scores (adjusted for age and sex)

	Depression	Anxiety	Stress	Resilience
Physical Health Summary Score	−0.29*	−0.44**	−0.36*	0.3*
Psychosocial Health Summary Score	−0.51***	−0.52***	−0.66***	0.3*
Total HRQOL	−0.47**	−0.54***	−0.61***	0.33*
Resilience	−0.52***	−0.65***	−0.35***	–

*p  < 0.05

**p < 0.01

***p < 0.001

Correlations between Cogstate *z*-scores and resilience were all small. However, moderate-to-strong negative correlations were identified between resilience and depression (*r* = −0.52, *p* < 0.001), anxiety (*r* = −0.65, *p* < 0.001), and stress scores (*r* = −0.35, *p* = 0.02). Moderate positive correlations were identified between resilience and HRQOL (*r* = 0.33, *p* < 0.02) and psychosocial health summary scores (*r* = 0.3, *p* = 0.04) (Table 6).

## Discussion

Long-term survival of individuals with complex CHD has dramatically improved over recent decades due to advances in surgical strategies and clinical care [2, 3]. Good long-term clinical outcomes are predominantly defined in terms of cardiac function and physiological health status; however, there is growing recognition of the cognitive [17–20] and psychological sequelae [4] experienced by this cohort which can impact physical and psychosocial health [4, 21, 46]. Associations between cognitive functioning and psychosocial factors in adults with complex CHD are poorly characterized. The current findings add to a growing body of literature that demonstrates a high prevalence of cognitive dysfunction in adults with complex CHD [18–20]. Cognitive functioning was correlated with socioeconomic factors; however, small correlations were observed between performance-based cognitive scores and self-reported psychosocial functioning. Greater resilience was strongly associated with lower psychological distress and higher HRQOL.

Common areas of cognitive dysfunction identified in the current sample included attention, working memory, psychomotor speed, and visual memory, that have similarly been reported in others adult CHD cohorts [18–20, 47, 48]. Elevated symptoms associated with ADHD were also frequent. Consistent with our previously reported findings, processing speed was slower in adults with a Fontan circulation compared with adults with biventricular complex CHD [20]. Adults with severe CHD had poorer verbal memory compared to those with moderate CHD. Importantly, our results suggest that adults with complex CHD may not recognize or report their cognitive challenges, highlighting the need for routine cognitive evaluation in this cohort to ensure adequate support is in place to optimize functional outcomes. Establishing clear referral pathways to neuropsychology services are essential. The use of screening tools may help to identify new or worsening cognitive and psychological dysfunction and assist in the referral process. Research investigating the sensitivity of screening measures such as self- and informant-questionnaires or computerized cognitive batteries in complex CHD cohorts are warranted to guide the application of these resources within the ACHD clinic. Global cognitive functioning was lower for individuals who were more socioeconomically disadvantaged. Similar findings have been demonstrated in younger complex CHD cohorts [22–24] and may reflect barriers to accessing neurodevelopmental screening, follow-up, and/or interventional services [49], differences in early-life environmental experiences and cognitive stimulation [50, 51], as well as cognitive heritability [52]. Hypothesized gene–environment interaction models suggest there is an interaction between cognitive-associated genetic predisposition and socioeconomic status that drives long-term cognitive outcomes [53–55]; however, these findings are variable and may particularly apply to families with higher socioeconomic status [56, 57]. Reduced cognitive functioning may limit employment opportunities and contribute to socioeconomic disparity in adulthood.

Currently, access to neuropsychological services for people with complex CHD in Australia is limited and costly [58], which compounds the high financial burden associated with having a complex cardiac condition [59]. Minimizing socioeconomic barriers that limit access to neuropsychological services for people with complex CHD is becoming a recognized health priority [60]. Higher socioeconomic status is considered a protective factor against cognitive decline in healthy aging adults [61, 62]. Early cognitive decline and dementia is predicted to be an impending issue for the growing adult CHD population [63, 64], and socioeconomic status may serve as an indicator for adults with complex CHD who may require closer monitoring.

Cognitive challenges in children with complex CHD are associated with lower academic performance and achievement [11, 14, 65, 66]. In the current cohort, lower global cognitive functioning and poorer attention and visual learning were identified in those who had lower educational attainment compared with those who had attained a university level degree. While we cannot determine whether lower cognitive functioning was a barrier to accessing higher level education in this sample, identifying cognitive challenges in people with complex CHD is vital to promote engagement with remedial academic services that may subsequently bolster higher level academic achievement and employment opportunities [67].

The prevalence of a diagnosed psychological condition in our adult complex CHD sample was similar to the general Australian population (41% versus 44%, respectively); however, a considerably greater number of participants had received a diagnosis of depression (33% versus 11% of Australian population [37]) which is consistent with reports from other adult CHD cohorts [68–70]. Elevated levels of psychological distress (i.e., depression, anxiety, and stress) impacted two-thirds of our cohort and correlated strongly with overall psychosocial functioning, suggesting that mental health challenges are negatively impacting social and occupational functioning. Greater resilience correlated with lower psychological distress and greater HRQOL, which has been reported in both child and adult CHD populations [71]. Therapies focused on optimizing psychological resilience in people with CHD are anticipated to have broad benefits for overall health and well-being [4, 71, 72]. Combined cognitive and psychological intervention may optimize functional outcomes [73].

Of the participants who had a lifetime diagnosis of a psychological condition, 44% had taken prescribed psychotropic medications at some point. Promisingly, 92% had received therapy from a psychiatrist or psychologist. While this indicates good access to services for those who have sought medical treatment, it also highlights the high service utilization and needs associated with psychological sequelae in adults with complex CHD. Given the relative socioeconomic advantage and geographical location of our current cohort of urban-dwelling adult complex CHD patients, the generalizability of this finding to the broader adult CHD population is likely limited. Socioeconomic disadvantage is recognized as a predominant barrier to accessing mental health services [74]. The current findings may not reflect people who live in more remote/rural areas who have been found to have poorer access to mental health services in Australia [74, 75]. Consistent with this, low utilization of other recommended clinical services, including cardiopulmonary exercise testing, has been reported in people with complex CHD in rural regions of Australia [76]. Access to clinical neuropsychology services for all adults with CHD is fundamental [4].

Somewhat surprisingly, correlations between severity of psychological distress and cognitive functioning in our cohort were negligible. In other clinical and ‘healthy’ cohorts, strong associations have been identified between severity of psychological distress and cognitive functioning [77–79]. Despite the high prevalence of psychological distress experienced in our current and other adult CHD cohorts [68, 70], our findings may suggest that reduced cognitive functioning observed in adults with complex CHD is predominantly due to organic neurocognitive dysfunction, i.e., the structural changes observed in the brain of people with complex CHD [80, 81]. Significant associations between reduced brain volumes and cognitive functioning have been demonstrated in both children and adults with complex CHD [20, 82–85]. Associations between altered white matter microstructure and poorer cognitive functioning have also been identified, however are not fully established [86–91]. Future research is required to determine the mediating impact of psychological distress on these associations. It is likely that cognitive outcomes are determined by a complex and dynamic interplay of neurobiological, physiological, and psychosocial factors that occur across the lifespan of people with complex CHD [92].

Correlations between cognitive functioning and patient-reported psychosocial health and HRQOL were also weak, which may be reflective of the discordance between self-reported cognitive functioning and performance-based cognitive outcomes demonstrated in our current cohort. In contrast, significant moderate correlations were observed between patient-reported physical health and cognitive functioning. The physical functioning scale on the PedsQL has a key focus on abilities relating to exercise and physical fitness (e.g., ‘*it is difficult for me to play sport or do exercise*’). A growing literature highlights associations between physical fitness, exercise, and cognitive functioning in healthy and clinical populations [93–98]. In the first study of its kind, Cooney et al. (2021) demonstrated significant associations between exercise capacity and sustained attention and adaptive functioning in people with a Fontan circulation [99]. Poorer exercise capacity in people with complex CHD may reflect worse physiology [100, 101] which possibly underpins associations with cognitive outcomes. Further research including detailed phenotyping is required to better understand associations between physical health and fitness and cognitive functioning in adults with complex CHD. However, evidence suggests that exercise may be a cost-effective and accessible intervention to optimize both physical and cognitive functioning in people living with complex CHD [102–104].

## Limitations

The current sample is relatively small and no demographically matched control participants were included. Overall, the sociodemographic profile of our cohort was generally higher than the general Australian population; however, this likely reflects the study location and recruitment strategies and may not be reflective of the sociodemographic status of adults living with complex CHD more generally. Despite this, a high proportion of our cohort demonstrated cognitive dysfunction and psychological distress, which may in fact underestimate the burden of these challenges across the adult complex CHD population more broadly.

## Conclusion

Adults with complex CHD are at risk for cognitive dysfunction and may not recognize or report their cognitive challenges. Poorer performance-based cognitive functioning was associated with lower socioeconomic resources. Elevated levels of psychological distress were common but associations with cognitive performance were small. Formal cognitive evaluation in this cohort is essential to ensure appropriate support is in place to optimize day-to-day functioning. Resilience was inversely associated with psychological distress and may be a modifiable, protective factor for reducing psychological symptoms and optimizing HRQOL.
